# Learner handover: Perspectives and recommendations from the front-line

**DOI:** 10.1007/s40037-020-00601-4

**Published:** 2020-08-18

**Authors:** Stephanie T. Gumuchian, Nicole E. Pal, Meredith Young, Deborah Danoff, Laurie H. Plotnick, Beth-Ann Cummings, Carlos Gomez-Garibello, Valérie Dory

**Affiliations:** 1 Institute of Health Sciences Education, McGill University, Montréal, Québec Canada; 2 Department of Medicine, McGill University, Montréal, Québec Canada; 3 Department of Pediatrics, McGill University, and Montreal Children’s Hospital, McGill University Health Centre, Montréal, Québec Canada; 4 Department of General Practice, Université de Liège, Liège, Belgium

**Keywords:** Assessment, Workplace-based assessment, Competency-based medical education, Learner handover, Forward-feeding

## Abstract

**Introduction:**

Current medical education models increasingly rely on longitudinal assessments to document learner progress over time. This longitudinal focus has re-kindled discussion regarding learner handover—where assessments are shared across supervisors, rotations, and educational phases, to support learner growth and ease transitions. The authors explored the opinions of, experiences with, and recommendations for successful implementation of learner handover among clinical supervisors.

**Methods:**

Clinical supervisors from five postgraduate medical education programs at one institution completed an online questionnaire exploring their views regarding learner handover, specifically: potential benefits, risks, and suggestions for implementation. Survey items included open-ended and numerical responses. The authors used an inductive content analysis approach to analyze the open-ended questionnaire responses, and descriptive and correlational analyses for numerical data.

**Results:**

Seventy-two participants completed the questionnaire. Their perspectives varied widely. Suggested benefits of learner handover included tailored learning, improved assessments, and enhanced patient safety. The main reported risk was the potential for learner handover to bias supervisors’ perceptions of learners, thereby affecting the validity of future assessments and influencing the learner’s educational opportunities and well-being. Participants’ suggestions for implementation focused on who should be involved, when and for whom it should occur, and the content that should be shared.

**Discussion:**

The diverse opinions of, and recommendations for, learner handover highlight the necessity for handover to maximize learning potential while minimizing potential harms. Supervisors’ suggestions for handover implementation reveal tensions between assessment-*of *and *for*-learning.

**Electronic supplementary material:**

The online version of this article (10.1007/s40037-020-00601-4) contains supplementary material, which is available to authorized users.

## Introduction

Competency-based medical education (CBME) models are gaining traction, particularly in postgraduate medical education (PGME). These models are characterized by their consideration for learner development and progress towards competency through the repeated assessment and monitoring of learners’ progress [1–3]. The developmental lens of CBME models and the accompanying assessment approaches have led to an increased focus on learner handover, or ‘forward-feeding’, where information about learners is shared across educational phases, supervisors, and/or clinical placements [4, 5].

While learner handover is well-aligned with the goals of CBME, we know little regarding the potential risks, benefits, or strategies for its successful implementation within CBME [5]. Current views regarding learner handover in medical education vary substantially [6–14]. Some suggest that learner handover may be beneficial and lead to: a) improved learning through more tailored education and support [8–12], b) improvements in a supervisor’s ability to assess learners across competencies [2, 8], c) fewer informal discussions of learners (i.e., ‘hallway talk’) [9, 11], d) increased assessor accountability [11], and e) increased patient safety through the early identification of weak or problematic learners [8, 10–12].

Others have raised concerns that learner handover would introduce bias into the assessment process [6, 7, 9, 10, 12, 13]. Specifically, discussions or documentation regarding the prior performance of learners may result in them being treated differently, labelled, or stigmatized by future educators [6, 7, 9, 10, 12, 13]. This, in turn, could result in self-fulfilling prophecies—where both learners and assessors behave in a way that aligns with the previous judgements of others [7]. Some argue that, depending on the content of the discussions and assessment reports, learner handover may violate a learner’s right to confidentiality and privacy [6, 7, 10, 12, 13].

To date, the literature largely reflects opinions held by educational leaders, including the directors of clinical courses and postgraduate programs and associate deans [6–10, 12–14]. The perspectives of clinical supervisors, who directly train and assess learners and represent the majority of faculty members involved in formal or informal learner handover, are less well described in the literature. Understanding the perspectives of clinical supervisors is critical given the key role these individuals will play in the successful (or not) implementation and execution of learner handover, and their tendency to operate independently based on their own values and beliefs [15, 16]. As such, we explored the opinions of (i.e., risks vs. benefits), experiences with, and recommendations for successful implementation of learner handover among clinical supervisors. We aimed to present the full range of beliefs held about learner handover and ideas for successful implementation generated by the participants in our study. We also explored the relationship between certain demographic characteristics of clinical supervisors and their support of learner handover. We were particularly interested in whether experience and clinical specialty, which may reflect exposure to different work cultures and practices, would influence their degree of support for learner handover.

## Method

Data used for this study were drawn from a survey-based questionnaire component of a larger experimental study investigating learner handover in PGME (findings from the experimental study are reported elsewhere [17]). All components of this study were approved by the Institutional Review Board of the Faculty of Medicine at McGill University (IRB#: A02-B08-17B).

### Participants and recruitment

The principal investigator (VD) contacted the directors of five large PGME programs (Family Medicine, Emergency Medicine, Pediatrics, Surgery, and Internal Medicine) at McGill University by email and invited them to circulate the recruitment email to their clinical supervisors. The program directors reported circulating our email to an estimated 700 supervisors (although this number does not account for potential duplicates, i.e. supervisors affiliated with multiple departments). Recruitment also occurred through presentations held at departmental meetings. The estimated time commitment was 30–40 min in total to complete the questionnaire and experiment.

### Procedure

Those interested in participating were provided with a link to the study (hosted through *LimeSurvey* [18]). Following consent, participants completed the experimental component of the study (reported in [17]) and the questionnaire (reported here). All participants who provided their email addresses were given a 100 Can$ (approximately 75 US$) Amazon gift card. All response data were kept in a separate file from participants’ identifying information.

### Questionnaire

The questionnaire included open-ended items (generating qualitative data) and closed-ended items (generating quantitative data) developed by the research team. Items were reviewed for relevance and clarity by the full research team, which includes several members from the target population of clinical supervisors. The questionnaire was not pilot tested. To limit participant cueing, we used five open-ended questions designed to gather narrative responses regarding participants’ experiences with, and perspectives on learner handover. The closed-ended items included one Likert-style item assessing a participant’s position on learner handover (“*Overall, what is your position regarding the sharing of information regarding residents’ previous performance across rotations?”*), with the following five response options: *Strongly Oppose, Somewhat Oppose, Neutral, Somewhat Favour, Strongly Favour. *The remaining closed-ended items comprised demographic questions targeting age, gender, clinical speciality, and the number of years spent supervising and assessing learners. The complete questionnaire is available as Appendix 1 of the Electronic Supplementary Material.

### Data analysis

#### Quantitative analysis

We computed frequencies for the closed-ended questions. We explored relationships between participants’ self-reported position on learner handover (i.e. Likert-style item) with age, years supervising, and years assessing using a Spearman correlation, and with participants’ gender and medical specialty using chi-square tests in IBM SPSS Statistics Version 21 for Mac OS X (IBM Corp, Armonk, New York).

#### Qualitative analysis

We approached our analysis from a stance of qualitative description, which favours staying close to participants words, with low inference categories, in order to capture reported perspectives of clinical supervisors on learner handover [19]. Qualitative description aligned with our goals and the data collection approach (fairly large sample, open-ended questionnaire items). Specifically, we used an inductive content analysis approach, informed by previous studies of stakeholder opinions [6, 9, 10, 12, 13], to analyze responses to open-ended survey items [20, 21]. In this approach, repeated ideas and key concepts are labelled, coded, and categorized, following a framework derived from both the data itself and from pre-existing theoretical knowledge of the topic under study [20–24]. We used *Atlas.ti* for Mac OS X (ATLAS.ti Scientific Software Development GmbH, Berlin, Germany) to facilitate coding.

In line with our post-positivist stance, we involved multiple team members in the coding process, recognizing that coders bring prior knowledge and beliefs to the table. In order to minimize the influence of individual bias, two independent coders (STG, NEP) familiarized themselves with the survey responses. For each open-ended survey question, the coders examined responses in batches of 10–20 respondents. Each coder independently broke down a participant’s answer into either one or multiple distinct units of meaning. Then, they assigned specific codes to every unit of meaning, and reviewed all the codes until they agreed on the best code(s) to use to represent each different unit of meaning. They applied this iterative coding process to all responses across the five survey questions. In cases where they could not reach consensus, they consulted a third coder (VD). Once they had established an initial coding scheme, the coders grouped independent codes into overarching categories and subcategories identified directly from the data and by drawing on existing literature [6–10, 12–14]. Once a complete round of coding and categorization was finalized, the third coder (VD) independently reviewed all of the assigned codes to ensure consistency in terminology and coding practice. Our research team discussed all codes and subcategories until we identified a final group of core categories. Such consensus-seeking aimed to leverage the different perspectives of team members and reduce the risk of misinterpretation.

We chose to examine the frequency of each code in order to explore how often particular opinions or concerns were mentioned and to facilitate comparison of code frequency with self-reported support of learner handover [24]. The quantification of qualitative data is possible when using a qualitative descriptive stance [20], when all participants are asked the same questions, and when the sample size is sufficiently large [25]. To do this, we computed the percentage of participants who mentioned each code, to gauge how common each mention of a code was within our relatively large (compared with most qualitative studies) sample [24]. To examine patterns of codes among participants with the same and differing positions on learner handover, we created a matrix with codes as rows, position on learner handover as columns, and the number of participants mentioning each code as cells.

## Results

### Participant characteristics

Seventy-two supervisors participated (participant characteristics are described in Tab. 1). One participant only completed the closed-ended questions. Response rates for open-ended items ranged from 93–99%, yielding 350 narrative responses out of a potential total of 360 (72 participants multiplied by five open-ended questions).

**Table 1:** Participant characteristics

Variable	Total (*N* = 72)
**Age (years)**	
Mean (SD)	46.2 (11.3)
Range	27–72
Respondents, *n*	71
**Gender**	
Male, *n* (%)	41 (57.8%)
Female, *n* (%)	29 (40.9%)
Other, *n* (%)	1 (1.4%)
Respondents, *n*	71
**Specialty**	
Internal Medicine, *n* (%)	22 (30.6%)
Family Medicine, *n* (%)	18 (25.0%)
Surgery, *n* (%)	12 (16.7%)
Emergency Medicine, *n* (%)	12 (16.7%)
Pediatrics, *n* (%)	7 (9.7%)
Other, *n* (%)	1 (1.4%)
Respondents, *n*	72
**Experience supervising (years)**	
Mean (SD)	16.6 (10.6)
Range	2–46
Respondents, *n*	72
**Experience assessing (years)**	
Mean (SD)	15.7 (10.8)
Range	1–42
Respondents, *n*	72
**Position on learner handover**	
Strongly oppose, *n* (%)	9 (12.5%)
Somewhat oppose, *n* (%)	15 (20.8%)
Neutral, *n* (%)	7 (9.7%)
Somewhat favour, *n* (%)	24 (33.3%)
Strongly favour, *n* (%)	17 (23.6%)
Respondents, *n*	72
Response rate for qualitative items #1–5*^a^	67–71 (93–99%)
Item #1—Benefits	71 (99%)
Item #2—Risks	71 (99%)
Item #3—Should be Shared	71 (99%)
Item #4—Should not be Shared	67 (93%)
Item #5—Experiences with Learner Handover	70 (97%)

^a^ Appendix 1 contains the complete wording for the five open-ended items

### Position on learner handover and relationships with demographic variables and codes

Positions on learner handover varied across participants on the Likert-style item ranging from *Strongly Oppose *to *Strongly Favour* (Tab. 1), with a majority in favour but one third of participants opposing learner handover. Position on learner handover was significantly associated with only two variables: supervisors with more experience in supervising and assessing learners were significantly more favourable towards learner handover (*r*_*supervising*_ = 0.25, *p* < 0.05; *r*_*assessing*_ = 0.29, *p* < 0.05).

### Findings from the open-ended survey items

Appendix 2 of the Electronic Supplementary Material includes a breakdown of all the codes, subcategories, and categories. In examining our matrix of code frequencies by level of support for learner handover, we found no major differences in the codes used across participants based on their level of support of learner handover; therefore, our main findings are presented collapsed across stated levels of support. The percentages reported below refer to the number of participants who mentioned a specific code at least once. The term ‘resident’ refers to postgraduate learners, ‘rotation’ to a clinical placement.

Conceptions of learner handover appeared to vary among participants, with some referring to a ‘learning plan’, suggesting a specific format and process of reporting, and others referring to the sharing of current end-of-clinical placement assessment reports without additional features. Most participants had no formal experience with learner handover but some described experiences of informal forward-feeding (e.g., conversations between supervisors), which they felt had led to positive and/or negative outcomes.

#### Benefits of learner handover

Three major subcategories representing potential benefits of learner handover were discussed by participants: (1) more tailored learning, (2) improvements to the assessment process (i.e., efficiency, learner monitoring), and (3) benefits to patient safety.

The benefit of learner handover mentioned by most (*n* = 50, 69%) of the participants in our study was the potential for more individualized learning: *“with earlier recognition of potential growth areas of the resident, the learning environment can be shifted to accommodate his/her learning needs” (P2)*.

Other reported benefits included enhancing the assessment process. Many participants (*n* = 34, 47%) mentioned how learner handover could improve efficiency: *“the faculty [could] spend less preliminary time in assessing the resident [so they] can get on with more specific training in needy areas” (P2). *Some participants (*n* = 21, 29%) also felt that it would improve assessment quality through the monitoring of learner progress. Others felt that it would generate more well-rounded assessments (*n* = 8, 11%), and reduce the risk of ‘failure to fail’ (*n* = 6, 8%) (although two participants, 3%, believed that it could increase this risk *“in a context where ‘forward feeding’ is permitted, why ‘fail’ someone or give someone a ‘borderline pass’ if we know that the problem can be passed on to someone else down the line to deal with it” (P27)).*

A few participants (*n* = 5, 7%) felt that it would be useful to ‘alert staff’ to weak learners requiring more oversight. A few others (*n* = 7, 10%) identified how the close monitoring of weak or problematic learners could improve patient safety.

#### Risks of learner handover

Participants’ primary concern was the potential for learner handover to lead to biased perceptions of learners (i.e., stigma, labelling) which, in turn, would have consequences for (1) the validity of subsequent assessments, (2) educational opportunities for learners, and (3) learner well-being.

Concerns about how learner handover could lead to labelling and stigma were raised by many participants (*n* = 33, 46%): “*a resident [might be] pigeonholed as being a negative trait (lazy, unprofessional). It biases the new supervisor. The resident does not feel like he gets a fresh start” (P16).* Most participants (*n* = 62, 86%) were concerned that sharing inaccurate information about a learner could bias subsequent assessments, impeding their validity: *“a bad evaluation in a previous rotation will likely affect the next clinician’s impression. The earlier evaluation might not be an accurate assessment (i.e. due to evaluator’s biases, due to one rotation of poor performance)” (P58).*

Some participants (*n* = 13, 18%) expressed how learner handover may negatively impact learner training and available educational opportunities. Specifically, concerns about halo effects, self-fulfilling prophecies, and confirmation bias were raised: *“strong residents tend to be let off easy whereas residents with difficulty will more likely be scrutinized” (P34).*

A few participants (*n* = 6, 8%) worried that biased assessments would “[*lead*]* to an environment where learners may fear that they have to be perfect because the information will be fed forward” (P22).*

Of note, potential legal challenges were raised by one participant.

#### Suggestions for how to implement learner handover

Participants provided diverse suggestions for implementing learner handover, which we categorized as: (1) procedural suggestions and (2) content-related suggestions:

- **Procedural: When and for whom should learner handover occur?**
  Some participants felt that sharing information would be helpful if a learner is struggling and would benefit from individualized help (*n* = 24, 33%) and if there were concerns about patient safety (*n* = 11, 15%).
  A few participants identified situations where they felt learner handover should not occur, including if a learner’s current assessment is being contested (*n* = 3, 4%), if there are potential legal implications (*n* = 2, 3%), and if the learner is on probation (*n* = 2, 3%). A few participants (*n* = 8, 11%) felt information should not be shared if there is ongoing interpersonal conflict between a resident and supervisor.
- **Procedural: How should learner handover be communicated and who should be involved in the process?**
  Some participants (*n* = 18, 25%) commented on how the information should not be shared with all staff, although a few participants (*n* = 4, 6%) held the opposite belief. One participant expressed that *“to reduce bias, it might be preferable if only some of the attending staff are aware of the past history—particularly anyone in a role-coaching situation. The other observers can be looking with a neutral eye” (P60). *A few participants (*n* = 4, 6%) stated that the program director and/or academic advisor should be involved as a core person in the receiving and relaying of this information. A few participants felt that learners should be involved in the process (*n* = 9, 13%) and that their assessments should only be shared if the learner consents (*n* = 10, 14%).
- **Procedural: How can learner handover be implemented in a fair way?**
  A few participants (*n* = 10, 14%) mentioned the importance of ensuring that the learner handover process is objective and rigorous as a way to reduce potential bias, improve assessment accuracy, and be fairly implemented. A few participants (*n* = 4, 6%) felt that all comments should describe behaviours and avoid moral judgements. One participant highlighted how supervisors need to *“embody the highest ethical and professional standards towards the use of this resource” (P33)*.
  A few participants (*n* = 4, 6%) believed the learner handover process should be accompanied by transparent communication and/or training *“to ensure both resident and attending know why this is being done” (P50)*.
- **Content: What information should be included?**
  Contradictory opinions related to sharing personal information about a learner emerged, with 10 participants (14%) against, and 2 (3%) for. These divergent views centred on concerns about confidentiality, the utility of providing personal information as a means to explain and understand performance, and whether these personal factors may influence learner performance on future rotations.

Participants disagreed on whether the forwarded information should include content related to learners’ weaknesses, strengths, or both. Many participants (*n* = 26, 36%) felt that handovers should contain information related to a resident’s weaknesses, for example: *“the details, specifics, or justifications for weak marks (or fails) are not pertinent to the next rotation but […] areas where the trainee and the supervisor need to focus are best shared” (P46).* Regarding a resident’s strengths, one participant described how *“it is also useful to feed forward information about residents who are strong, as this helps to know when residents can be given extra trust/responsibility and to challenge these residents to take on more advanced roles” (P47)*. However, concerns about the sharing of a learner’s strengths leading to halo effects or supervisor and/or resident complacency were also raised.

## Discussion

Learner handover is a controversial topic in medical education [5, 7, 8, 14]. Studies about stakeholder views towards learner handover have primarily focused on educational leaders [6, 9, 10, 12, 13], with only one recently published study including the perspectives of medical students and residents [10]. To our knowledge, our study is one of the first to explore the perspectives of clinical supervisors. Recognizing and reacting to supervisors’ perspectives in the design and implementation of educational interventions is critical to reducing the gap between intended and enacted interventions which rely on supervisors’ behaviours [15].

The perspectives of frontline supervisors in our study were divided, with a slight majority in favour but a third opposing learner handover. This is similar to studies of educational leaders such as clinical course (clerkship) directors [6]. Our participants also mentioned a similar range of potential risks and benefits of learner handover within medical education programs [6, 9, 10, 12]. Participants described two main benefits: 1) providing opportunities for more tailored learning, and 2) improving the overall assessment process through increased efficiency and better longitudinal monitoring. Participants discussed how these benefits could ultimately improve patient safety. The largest concern regarding learner handover was that it may bias a supervisor’s perception of a learner which, in turn, could impact the validity of future assessments, the educational opportunities afforded to learners, and the general well-being of learners.

While previous publications emphasized the division between those in favour of learner handover and those against it [6–9], we found that regardless of their degree of support for learner handover, clinical supervisors articulated a similar range of advantages and disadvantages of learner handover. This suggests that those who oppose and those who support learner handover largely share common beliefs, although perhaps they weigh the benefits and risks differently.

Supervisors provided a broad range and occasionally conflicting recommendations for potential implementation. In particular, some participants felt that struggling learners would benefit most from learner handover, whereas others recommended avoiding learner handover in such cases due to concerns about the defensibility of progress-related decisions. This paradox—struggling learners being both the priority target and the most at risk of the potential negative consequences of learner handover—highlights existing tensions in workplace-based assessment systems, specifically between assessment-*for-*learning and assessment-*of-*learning [26, 27]. The main potential benefits of learner handover fit soundly within an assessment-*for*-learning framework, commonly espoused by CBME [3, 28], where the goal is to leverage the educational effects of assessment, specifically through the ‘catalytic effects’ of feedback, and to continuously provide learners with the support necessary to achieve target competencies [27, 29]. However, most PGME programs are adopting a programmatic approach to assessment and make use of the same workplace-based assessments for assessment-*for* and *of*-learning. In order for such programs of assessment to also be fit for a purpose of assessment-*of*-learning, assessments must meet stringent validity and reliability criteria, which include minimizing or mitigating bias [26, 27, 30].

Govaerts and colleagues suggest that tensions in opinions related to assessment should not always be resolved by ‘either-or thinking’ (e.g. either implementing learner handover to maximize learning or prohibiting it to avoid bias) [26]. Rather, the tension should be approached in a way that seeks to optimize implementation to maximize the benefits and minimize the harms. Our participants made several recommendations that suggested they were approaching the issue of learner handover through such a lens. For example, participants emphasized the need to ensure a rigorous and objective process, and to carefully determine who should and should not be privy to forward-fed information. These key issues have in fact been considered by the educational leaders who have designed learner handover processes that have already been implemented. In particular, existing models of learner handover limit information-sharing to specific individuals (such as educational supervisors in the United Kingdom for example) [31] and/or groups of individuals (such as competency committees in North America) [32].

Our findings suggest that clinical supervisors, like educational leaders, recognize both the potential risks and benefits of learner handover. Therefore, initiatives designed to win over the hearts and minds of those reluctant clinical supervisors by informing them of the wealth of potential benefits of learner handover are unlikely to bear fruit. Instead, educational leaders implementing learner handover should engage all stakeholders in designing learner handover processes with a lens towards optimization. ‘Polarity thinking’ provides a framework to collaboratively define action steps that will increase the likelihood of positive outcomes and early warning signs that will alert to the risk of negative outcomes [26].

### Limitations

The use of free-text survey items in medical education has been criticized for lacking depth, richness, and rigor as they often do not contain ‘context, personal meaning, emotional and social nuances and layers of detail’ [33, p. 347]. Further, our open-ended items were developed iteratively by our research team, but were not pilot tested. We adopted a methodology of qualitative description, which is purposeful and often used to obtain straightforward answers to questions that can then be used to inform practice and policy [19]. Given that research on learner handover is limited, we aimed to simply gather a range of opinions towards learner handover and provide context for the responses to the closed-ended survey responses. Future studies, using interviews or focus groups, could examine supervisors’ beliefs about learner handover in more depth. We also recognize some of the opinions reported were mentioned by a small number of participants in this study. However, our goal was to not only capture the most popular opinions regarding learner handover but to identify the full range of beliefs about learner handover and ideas for successful implementation. The response rate at our institution was low (an estimated 10%) which may mean that our findings are representative of a specific type of supervisor thus limiting our ability to generalize our findings to a broader body of clinical supervisors. Participants agreed to commit approximately 30 min to the study as a whole, which may have led us to recruit supervisors who were less busy or who had an interest in medical education. Compared with a shorter study at the same institution, but in different clinical disciplines [34], we found that our participants were more likely to have growth mindsets (99% versus 88% for clinical reasoning, 71% versus 35% for empathy—data reported elsewhere [17]) than participants in the shorter study, suggesting a potential selection bias. Our sample was nonetheless diverse in terms of its demographic characteristics and its positions on learner handover. Further, our sample included clinical supervisors from one Canadian institution, and our findings may be influenced by the institutional policies and culture present at this institution and may not generalize well to other learning contexts. Specifically, at the time of this study, our institution had a strict policy prohibiting learner handover amongst clinical supervisors. As such, only a few participants in our study reported experiences with learner handover and, among those who did, their experiences were informal. Given the novelty of learner handover, especially within Canadian medical education programs, we did not anticipate being able to collect detailed accounts of perspectives on personal experiences with learner handover. Thus, these findings provide baseline data on the range of perceptions that must be considered in preparing clinical supervisors to take on learner handover in a meaningful manner. Our institution has implemented Focused Learning Experiences (FLEX) periods tailored to learners’ educational needs involving coaching and role mentoring, thus working towards normalizing the occasional need for remediation. This may have also influenced our participants’ perspectives on the developmental nature of competence and the appropriateness of learner handover.

### Future directions

While understanding supervisor perspectives is critical to successful implementation, discussions regarding learner handover would benefit from robust evidence regarding the observed benefits and harms of learner handover as well as factors influencing their likelihood and extent. The majority of available experimental studies can be found within the educational, social, and organizational psychology literature [5, 35–38], with a small, but growing, literature in health professions education with mixed results [17, 39]. Further studies are needed to understand the potential positive and negative impacts of learner handover in medical education settings. The evidence should then be used to inform and engage stakeholders, including clinical supervisors, in collaboratively designing how learner handover should be implemented and carefully monitored for maximal benefits and minimal harms.

## Caption Electronic Supplementary Material

Full questionnaire (Appendix 1) and the final codebook with sample quotations, number of code mentions, and proportion of participants mentioning (Appendix 2)
