# Supplementary material for: Learner handover: Perspectives and recommendations from the front-line
Source: Perspect Med Educ. 2020 Aug 18;9(5):294–301. doi: 10.1007/s40037-020-00601-4 (PMC7550510; doi:10.1007/s40037-020-00601-4)
Supplement: Supplementary file 1 — Full questionnaire (Appendix 1) and the final codebook with sample quotations, number of code mentions, and proportion of participants mentioning (Appendix 2) [file 40037_2020_601_MOESM1_ESM.docx]

**Appendix 1.** Full questionnaire with demographic and open-ended survey questions pertaining to participants’ experiences and opinions of learner handover

**Demographic Questionnaire**

1. Please indicate your age (years):
2. Please indicate your gender:

- Male
- Female
- Other

1. Please indicate your specialty:

- Emergency Medicine
- Family Medicine
- Internal Medicine
- Pediatrics
- Surgery
- Other: please specify

1. For how many years have you been involved (as an attending physician or as a resident) in **supervising** medical students and/or residents in the clinical setting?

1. For how many years have you been involved (as an attending physician or as a resident) in **assessing** medical students and/or residents in the clinical setting?
2. Overall, what is your position regarding the sharing of information regarding residents’ previous performance across rotations?

- Strongly oppose
- Somewhat oppose
- Neutral
- Somewhat favour
- Strongly favour

**Open-ended survey questions pertaining to participants’ experiences and opinions of learner handover**

1. What do you think are the potential benefits of sharing information regarding residents’ previous performance across rotations?
2. What do you think are the potential risks of sharing information regarding residents’ previous performance across rotations?
3. In your opinion, should such information ever be shared across rotations? If so, in what circumstances and how?
4. In your opinion, are there ways or circumstances in which such information should never be shared?
5. Have you had any specific experiences with information sharing across rotations?

**Appendix 2.** Final codebook with individual codes, subcategories, categories, total code mentions, proportion of mentions, and example quotations

| Benefits of Learner Handover  *Q: What do you think are the potential benefits of sharing information regarding residents' previous performance across rotations?* | | | |
| --- | --- | --- | --- |
| Category, Subcategory, & Codes | **Example Quotation** | **Total # of Code Mentions** | **N (%) of participants who mentioned code** |
| *Subcategory: More Tailored and Targeted Learning* | | | |
| Helps identify rotation specific issues | “If the resident did "badly" in actual rotation the residents' previous performance across rotations (other subspecialties, similar units in other hospitals) can rise some issues specific to actual rotation in order to be fixed (unit environment, relationships in the unit, individual personalities of team members; personal issues of the trainee). If the trainee's issue is still persisting across rotations it can help to find appropriate ways for solving them.” | 1 | (1) 1% |
| Individualized learning | “It allows new teachers to pay attention to past difficulties and work on them. It allows for greater continuity in teaching objectives and allows the learner to demonstrate development of those skills.” | 83 | (50) 69% |
| Leads to learner improvement | “Improvement.” | 1 | (1) 1% |
| *Subcategory: Supports Learners and Fosters Engagement* | | | |
| Communicates to learners that they are supported | “Will send a message to the resident that […] the institution cares in the sense that 'we want you to succeed' and we will work to make sure that this happens (the hidden message there is that the supervisors are thinking beyond the confines of the rotation....it is not like they wash their hands of the resident upon completion of the rotation).” | 4 | (3) 4% |
| Encourages self-direction | “It allows teachers to identify areas to improve or try new ways to engage the resident in self-reflection.” | 3 | (3) 4% |
| *Subcategory: Improvements to the Assessment Process* | | | |
| Better than informal sharing | "Objective information may be better than inferred information or gossip.” | 1 | (1) 1% |
| More well-rounded assessments | “Without any forward feeding, we might miss key gaps that we won't necessarily be fortunate enough to see in limited exposures with the trainee.” | 8 | (8) 11% |
| Efficiency | “The faculty spends less preliminary time in assessing the resident and can get on with more specific training in needy areas.” | 44 | (34) 47% |
| Fosters collaboration among supervisors | “Useful when seeing trainees in small snippets and can validate impressions/findings with colleagues I trust, and build a plan to help together that we all help implement (so the remediation is not all on one person's shoulders). Very good for the resident, and reassuring and supportive for the attendings.” | 1 | (1) 1% |
| *Subcategory: Monitors Learners Closely* | | | |
| Reduces failure to fail | “Most people are afraid to give bad evaluations, and tend to give residents the benefit of the doubt, assuming it may not be always like that and it is specific to that specific rotation, specific period of time (resident undergoing personal difficulties?). But if we know it happened before, especially if noted on more than one occasions, than it allows evaluators to feel confident that this is a recurrent process, the norm rather than the exception.” | 6 | (6) 8% |
| Tracks progress | “It allows for greater continuity in teaching objectives and allows the learner to demonstrate development of those skills.” | 24 | (21) 29% |
| Alerts staff of weak learners | “Alerts staff if a learner needs to be observed or not given a high amount of responsibility without verifying their abilities first.” | 5 | (5) 7% |
| *Subcategory: Benefits to Patient Safety* | | | |
| Improves patient safety | “If patient safety is at issue then the benefits might be to make the staff pay more attention to details in patient care (ie if the resident can't really be believed or trusted).” | 8 | (7) 10% |
| Risks of Learner Handover  *Q: What do you think are the potential risks of sharing information regarding residents' previous performance across rotations?* | | | |
| *Subcategory: Biases Assessment* | | | |
| Biases the assessment process (general) | “Introducing bias to future evaluators may result in non-objective assessment.” | 96 | 62 (86%) |
| Assessments made by respected mentors may be more trusted | “Evaluations by well-respected mentors are given more weight” | 1 | (1) 1% |
| Inexperienced mentors may give more biased assessments | “Bias, especially with inexperienced mentors.” | 1 | (1) 1% |
| Short exposure to learner | “Introduce a bias in our perception of this resident, especially if we don't work with that resident for a long period of time.” | 1 | (1) 1% |
| Impacts the supervisor’s perception of the learner | “Once residents are labelled as deficient, this label can be particularly difficult to shake as it may generate confirmation bias in others.” | 44 | 33 (46%) |
| Increases failure to fail | “By knowing that our evaluation has an impact on their future residency training I guess we tend to be more generous if we know they are ‘on the edge’ of going to probation.” | 2 | 2 (3%) |
| *Subcategory: Impacts Learning* | | | |
| Harm to learner education | “learning opportunities are not provided in an unbiased way for the resident.” | 15 | 13 (18%) |
| Undermines self-direction | “Residents can resign themselves to be stuck in a certain mode.” | 3 | 3 (4%) |
| *Subcategory: Impacts Learner Well-being* | | | |
| Affects the learner’s environment | “Learner increased fear of having made a past "mistake" - making every learning environment less "safe," leading to an environment where learners may fear that they have to be perfect because the information will be fed forward.” | 7 | 6 (8%) |
| Impacts learner well-being (general) | “Residents can also feel a lot more pressured/stressed if they know that a new attending already has information about them (ie- if they did not do well on one rotation, they might feel they are negatively being judged from the get-go in the next rotation).” | 3 | 3 (4%) |
| Biased perceptions may impact learner | “Residents' perception that they are not being observed and judged objectively.” | 11 | 10 (14%) |
| *Subcategory: Other Risks* | | | |
| Defensibility (i.e., legal issues) | “there is a risk that decisions to give a 'Failing' grade will be more difficult to defend in a court of law.” | 1 | (1) 1% |
| Circumstances for Implementation: Procedural recommendations and content suggestions  *Q’s: (1) In your opinion, should such information ever be shared across rotations? If so, in what circumstances and how? (2) In your opinion, are there ways or circumstances in which such information should never be shared?* | | | |
| Category: Procedural Recommendations | | | |
| *Subcategory: What type of learner should it be done for?* | | | |
| Learner handover should be conducted for every learner | “This does not only need to be done when a resident is experiencing difficulty (though should be done in these cases in particular), but can also be for every resident so we can help them grow always.” | 1 | (1) 1% |
| Must be a case-by-case approach | “For the performance of basic clinical skills, in the absence of professional, mental health and religious accommodation issues, forward feeding is generally not necessary and, I think, not ideal. It must be a case by case approach.” | 5 | (4) 6% |
| Learner handover should only be done in extreme or unusual cases | “Not as a routine but in extreme or unusual circumstances and only when it has been thoroughly investigated discussed and authorized by a promotion committee.” | 4 | (3) 4% |
| If the learner needs individualized help | “If a learner needs more exposure to develop a yet undeveloped or underdeveloped competency.” | 24 | 24 (33%) |
| *Subcategory: Why should learner handover be done?* | | | |
| To identify patterns and recurrent issues | “At least somebody, who is objective, should compile the assessments and look if there is any pattern.” | 2 | (2) 3% |
| Patient safety | “if prior performance evaluation identifies problems that could seriously endanger patients, e.g. with seriously neglectful or harmful care.” | 13 | 11 (15%) |
| Should only be used to assess overall learner progress | “in the setting of an academic advisor reviewing the overall progress of a resident.” | 2 | (2) 3% |
| *Subcategory: When should learner handover reports NOT be shared?* | | | |
| If the learner’s assessment is being contested | “If the evaluation is being contested, it should not be shared - the confidence of the evaluation is being put into question.” | 3 | (3) 4% |
| If there are potential legal implications | “If there are medico legal implications and divulging the information could compromise the legal process.” | 2 | (2) 3% |
| If it risks putting the learner in an awkward position | “if it would breach confidentiality for the resident.” | 1 | (1)1% |
| If there is ongoing interpersonal conflict | “If the problems are the results from interpersonal issues (i.e. the trainee and supervisor did not ‘get along’).” | 8 | (8) 11% |
| If the learner is on probation | “If the learner is being put on probation, the specific evaluations should not be carried over.” | 2 | (2) 3% |
| *Subcategory: When should it be done?* | | | |
| Should not be shared without support available for learner | “if it would breach confidentiality for the resident or put them in an awkward or difficult situation without support.” | 1 | (1) 1% |
| If the next supervisor has that learner for a long period | “If the clinical supervision will be for a longer period of time, such as in CTU (2-4 weeks of exposure), or during longitudinal clinics but I would avoid sharing information when the exposure is limited to a short period of time (e.g. 1 week).” | 1 | (1) 1% |
| Information should be shared after the future assessment is complete | “Perhaps learning about previous rotations at the end of the rotation, before entering the final evaluation would be best to avoid potential risk as outlined above.” | 2 | (2) 3% |
| Information should be shared at the beginning of the rotation | “If it happened already more than once or something potentially really problematic already happened, then for sure before the next rotations would be best to ensure close evaluation of these specific concerns are observed and addressed.” | 1 | (1) 1% |
| *Subcategory: Who should be involved in the process?* | | | |
| Learner handover should not be shared with all staff members | “To reduce bias, it might be preferable if only some of the attending staff are aware of the past history - particularly anyone in a role-coaching situation. The other observers can be looking with a neutral eye.” | 23 | 18 (25%) |
| Learner handover should be shared with/by academic advisors | “Someone such as a mentor or a program director would be better suited to contextualizing the evaluations within the overall arc of the residents progress through their program.” | 5 | 4 (6%) |
| Learner handover should be shared with/by supervisors (e.g., attendings, assessors) | “I think this should be done between supervisors although not widely disseminated among all evaluators.” | 5 | 4 (6%) |
| Assessments should only be shared if the learner requests/consents | “It should be shared across rotations if and only if the learner is in agreement with the learning plan and with sharing the learning plan.” | 16 | (10) 14% |
| Learner handover should involve learners in the process | “With the trainee's consent and involvement. The trainee should be encouraged to articulate what "problems" they need to work on, and where they are doing well and would like to excel to the attending, who can validate this with information from previous rotations.” | 10 | (9) 13% |
| Learner handover should not be shared with the learner | “I personally would not use the 'leaning contract model'. I would not share the above detailed recommended strategy with the resident. I think it losses its effectiveness if shared a priori.” | 1 | (1)1% |
| Learner handover should be shared with learners | “Should be shared to residents, not to other staff attendings.” | 1 | (1) 1% |
| *Subcategory: How should learner handover be communicated?* | | | |
| Assessments should only be shared between similar rotations | “It should not be shared if the information is about performance specific to a certain rotation and not applicable to other rotations.” | 3 | (3) 4% |
| Should be shared anonymously | “Previous evaluations should maybe not reveal evaluator.” | 2 | 1 (1%) |
| Learner handover information must be kept confidential | “Specific comments from previous rotations should remain confidential.” | 4 | (3) 4% |
| Should be communicated via email or verbally | “If major concerns in trustworthiness, skills not at level - next staff should be made aware - verbal communication or email.” | 1 | (1) 1% |
| Information should never be shared informally (i.e., casually, gossip) | “Should never be shared casually in the hallways or in front of the resident`s colleagues. | 2 | (2) 3% |
| Information should not be anonymous | “Anonymous and context-free information should not be shared.” | 1 | (1) 1% |
| Information should not be shared electronically | “I think forwarding a written evaluation electronically should be discouraged.” | 1 | (1) 1% |
| *Subcategory: How can learner handover be implemented in a fair and equitable way?* | | | |
| Faculty using learner handover should be trained in the process | “If the faculty members have not been adequately trained in understanding the risks and benefits of forward feeding, the information should not be shared.” | 5 | (4) 6% |
| Learner handover should be normalized | “It should be normalized.” | 1 | (1) 1% |
| Staff should be trained on how to give constructive criticism | “Staff should be trained on constructive criticism.” | 1 | (1) 1% |
| Learners and staff need to be informed about the reasons for doing learner handover | “I think it should - overall better for trainee learning, but need to ensure both resident and attending know why this is being done.” | 1 | (1) 1% |
| Learner handover must be monitored carefully | “You must be careful who receives that information and what will be done with it.” | 2 | (2) 3% |
| Must be a standardized process | “It should be standardized and ideally include context-, illness-, and complexity-specific information when available. Also, evaluations shared between the same type of rotation (e.g. CTU) may be more useful than sharing information from disparate experiences e.g. ambulatory clinic evaluation to the ICU rotation.” | 2 | (2) 3% |
| Must be objective and rigorous | “It needs to be very concrete and objective. The way evaluations are done now are too subjective to make them useful and there is a real risk of labelling someone which can be difficult to shake.” | 11 | (10) 14% |
| Learner handover should be assessed using a separate form | “A specific standardized format separate from the assessment tool may be appropriate that highlights the areas of clinical competence and suggested areas of development.” | 1 | (1) 1% |
| Learner handover should be fair | “I think it can be detrimental to the resident if the information shared is an evaluation that doesn't fairly represent the resident.” | 5 | (5) 7% |
| Supervisors must remain professional and ethical during FFA | “Respect and discretion are critical.” | 2 | (2) 3% |
| Category: Content-Related Suggestions | | | |
| *Subcategory: What information should be included?* | | | |
| Must be comprehensive | “Such a strategy (i.e. of feeding forward) will really only be beneficial, I think, if it is done in a rigorous and comprehensive fashion .....for example, all aspects of the performance, not only the gaps, must be shared with the future supervisor.” | 3 | (3) 4% |
| Should be shared with context | “I believe context should always be included so as to inform about potential variables affecting resident performance.” | 4 | (3) 4% |
| Should include assessment across the whole rotation | “I believe evolution from beginning to end of rotation is a must as a resident can start out poor but finish strong.” | 1 | (1) 1% |
| Should include behavioural comments not moral judgments | “Comments should be behavior based, not moral judgements on the person. (comment that resident showed up late for his clinics 2 days per week rather than say he is lazy and not hardworking).” | 4 | (4) 6% |
| Should include future work goals and plans | “Comments should be made on what goals and plans have been put in place previously to work on problem areas.” | 3 | (3) 4% |
| Should include personal information | “A resident performs poorly because of a transient trigger that is now passed. For example, if a resident has just lost a close family member or has a personal situation that has led to a dip in performance, this should be fed forward to the training program and program director.” | 2 | (2) 3% |
| Should only include constructive comments | “Information should not be negative or prejudicial but put in a constructive way, e.g. Dr. X would benefit from observation in an acute care setting, or giving bad news, etc.” | 3 | (3) 4% |
| Identified strengths should be shared | “It is also useful to feed forward information about residents who are strong, as this helps to know when residents can be given extra trust/responsibility and to challenge these residents to take on more advanced roles” | 2 | (2) 3% |
| Share information on clinical reasoning skills (e.g., judgment) | “Future rotations should be given warning that a learner needs more supervision. This includes poor reasoning and judgement.” | 1 | (1) 1% |
| Share information on professionalism skills (e.g., trustworthiness) | “I think info should only be shared if there is a professionalism concern.” | 6 | (5) 7% |
| Share information on learner areas of concern & weakness | “The only information that I think might be useful for the residents if shared across rotations is if there was a major weakness that affects the resident's ability to become a clinician and it is important that the resident is able to overcome the weakness that was observed in the rotation.” | 26 | 26 (36%) |
| Must be followed by a tailored response plan | “In my opinion, the person who passes on the information also has an obligation to recommend specific remediation actions.” | 1 | (1) 1% |
| *Subcategory: What information should not be included?* | | | |
| Do not share information on fixed qualities | “When it isn't felt to be something that is readily changed.” | 2 | 2 (3%) |
| Assessments should not include previous marks | “The most important things to share would be the "things to improve or to work on", without necessarily a mention as to the mark attached to the previous evaluation.” | 1 | (1) 1% |
| Identified strengths should not be shared | “If a resident is performing well overall, to carry over the information from one rotation to another is neither beneficial or detrimental (Possible detriment could be complacency on the resident's part).” | 1 | (1) 1% |
| Personal information should not be shared | “Information relating to non-CanMEDs domains should not be shared e.g. if a resident was thought to perform poorly due to personal issues (illness, relationship issues, etc.).” | 10 | 10 (14%) |
| Previous failures or weaknesses should not be shared | “We should not know about their previous failures or borderlines.” | 4 | 4 (6%) |
| Should not share all information (i.e., if learner does one wrong thing) | “The details, specifics or justifications for weak marks ( or fails) are not pertinent to the next rotation.” | 5 | 5 (7%) |
| Should not be shared if the resident issue is resolved | “If the event happens under punctual circumstances that will likely not happen again. If the resident showed us enough improvement so we feel more secure” | 4 | (3) 4% |
| Participants’ Experiences with Learner Handover.  *Q: Have you ever had any specific experiences with information sharing across rotations?* | | | |
| *Category: Had informal learner handover experiences that confirmed benefits* | | | |
| Informal learner handover confirmed proposed benefits | “I have found that being "warned" in advance about poorly performing trainees has helped me to then spend a bit more time with them and try to see how I could be helpful.” | 15 | 15 (21%) |
| Informal learner handover led to more tailored learning | “It was used to improve resident’s surgical skills. resident was channeled to do more procedure to improve his skills and it happened.” | 7 | 7 (10%) |
| Informal learner handover helped track learner progress | “It has been done more in a manner to monitor the resident to determine if they are weak candidates or if they were unfairly evaluated during previous rotations.” | 1 | (1) 1% |
| Sharing led to collaborations among staff | “I have shared info I have felt useful to the staff coming on service: it helps specifically re expertise/safety with ICU procedures, safety during night shifts, and interactions with nurses etc.” | 1 | (1) 1% |
| *Category: Had experiences with learner handover that confirmed risks* | | | |
| Learner handover led to biased perceptions of learner | “Some residents have told me that once an impression is formed by preceptors in a longitudinal training program, especially in a promotions committee, they continue to be labelled that way and it is difficult to change minds.” | 7 | 7 (10%) |
| Harmed a learner’s education | “Unfortunately, many hallway conversations do provide information about residents and may negatively impact their performance when they start a new rotation.” | 4 | 4 (6%) |
| Affected learner well-being | “Mostly word to mouth. For myself has not created any problems but have seen friends/colleagues being “black listed” and how difficult and how much distress it creates.” | 1 | (1) 1% |
| No learner handover led to increased failure to fail and impacted patient safety | “There was one resident that I wish I had heard more about, I would have (appropriately) failed her when I thought she was unsafe. As it stands, I passed her, as did everyone else, and she is probably out somewhere being a bad doctor.” | 1 | (1) 1% |
| No learner handover impacted assessment efficiency and tailored learning | “It is very upsetting to a supervisor to learn that the deficiencies he or she has identified were well known from previous rotations.” | 2 | 2 (3%) |
| Sharing learner handover led to gossip | “There is gossip amongst attendings (and sometimes this gossip makes the rounds of the nurses) about specific mistakes that residents make or generalization about their character.” | 1 | 1 (1%) |
| Sharing was unhelpful | “certainly, and usually in a biased and unproductive manner.” | 1 | 1 (1%) |
| *Category: General experiences with learner handover* | | | |
| Shared information at the end of rotation | “yes, regularly we got gross evaluation of the resident overall skills, strength and weakness. It’s mostly shared at the end of the rotation when we evaluate the resident to compare our opinions with the other centers.” | 1 | (1) 1% |
| Shared by an academic advisor | “Only in a longitudinal review as an academic advisor.” | 1 | (1) 1% |
| Shared by the program director | “We have monthly faculty meetings in our clinic to evaluate the residents. During these meetings, the program director will sometimes provide further information about a particular resident in their recent rotations but we do not do this for all the residents.” | 3 | 3 (4%) |
| Shared information formally | “When I was a program director I saw all the evaluations – I found myself having to guard against the issues above.” | 6 | 6 (8%) |
| General experiences sharing | “Staff also talk amongst themselves informally.” | 34 | 34 (47%) |
| Learner handover assessment was not shared with learner | “I have seen staff discuss about residents and their evaluations informally. However, I believe this was done without the resident's knowledge.” | 2 | 2 (3%) |
| Information was shared in person | “The information was shared in person.” | 1 | 1 (1%) |
| Shared info about at-risk learners | “Only when the student is on probation.” | 8 | 8 (11%) |
| Information was shared over the phone | “The information was shared in person and over the phone.” | 1 | 1 (1%) |
| Sharing was used to determine fairness of previous evaluation | “It has been done more in a manner to monitor the resident to determine if they are weak candidates or if they were unfairly evaluated during previous rotations.” | 1 | 1 (1%) |
| Sharing occurred within rotations | “Not across but within – e.g., at handoff on inpatient weeks - ass along my impressions/evals of the performance to the next staff.” | 2 | 2 (3%) |
| Insufficient information was shared | “Yes, in cases of "remedial" rotations- unfortunately, the issues requiring "remediation" were never identified so could not be assessed or corrected in any formal way.” | 1 | 1 (1%) |
| Disagreed with previous assessment | “Yes, many times; usually helpful, but sometimes I disagreed w prior assessment (please note I work in a sub-specialty and as such the prior rotation may be very similar but at a different site, so usually quite helpful).” | 1 | 1 (1%) |
| Shared information informally | “Information sharing occurs only in an informal way in my experience.” | 2 | 2 (3%) |
| *Category: No experiences with learner handover* | | | |
| No experiences with learner handover (informally) | “We do not have any information sharing across rotations.” | 25 | 24 (33%) |
| No experiences with learner handover (formally) | “I have never been shown an ITER or evaluation from a previous rotation.” | 4 | 4 (6%) |
| *Category: Barriers to learner handover* | | | |
| Learner handover is forbidden | “We all do it but may not always admit it openly as we have been told that it is ‘forbidden’”. | 1 | (1) 1% |
| Weak residents did not want learner handover | “Most residents didn't want this to be done when offered in the past (Esp. residents with multiple weaknesses).” | 1 | (1) 1% |
| *Category: Facilitators to learner handover* | | | |
| Strong residents welcomed learner handover | “Strong residents often welcomed feeding forward.” | 1 | (1) 1% |
| General opinions voiced towards learner handover | | | |
| No benefits of learner handover | “I do not feel that there is much benefit for the resident to have his/her result shared with supervisors in various rotations.” | 2 | 2 (3%) |
| Learner handover should not always be shared | “Yes.” | 2 | 2 (3%) |
| Learner handover does not fit within a CBME approach | “From a competency-based perspective, the need to share information should not be important.” | 1 | 1 (1%) |
| Learner handover assessments should not be shared | “No. Each evaluation by individual assessors should be done independently.” | 20 | 15 (21%) |
| Unsure about whether learner handover should occur | “I have mixed feelings about this.” | 7 | 6 (8%) |
| Benefits of learner handover outweigh the harms | “Yes. I believe supervising attendings frequently reach the same conclusions about residents even without knowledge of what others have said in the past. As such, I think the benefits of feeding forward information is more helpful than harmful.” | 1 | 1 (%) |
| Learner handover is valuable/useful | “I believe that educational hand-over is a good thing overall.” | 4 | 4 (6%) |
| Learner handover assessments should be shared | “I do believe there is a place for some of the information to be shared.” | 40 | 35 (49%) |
